# Isotope-Labeled RNA Building Blocks for NMR Structure and Dynamics Studies

**DOI:** 10.3390/molecules26185581

**Published:** 2021-09-14

**Authors:** Lukasz T. Olenginski, Kehinde M. Taiwo, Regan M. LeBlanc, Theodore K. Dayie

**Affiliations:** 1 Center for Biomolecular Structure and Organization, Department of Chemistry and Biochemistry, University of Maryland, College Park, MD 20742, USA; lolengin@umd.edu (L.T.O.); ktaiwo@umd.edu (K.M.T.); Regan_LeBlanc@vrtx.com (R.M.L.); 2 Vertex Pharmaceuticals, 50 Northern Avenue, Boston, MA 02210, USA

**Keywords:** stable isotope, enzymes, nucleotides, NMR spectroscopy, RNA, structure, dynamics

## Abstract

RNA structural research lags behind that of proteins, preventing a robust understanding of RNA functions. NMR spectroscopy is an apt technique for probing the structures and dynamics of RNA molecules in solution at atomic resolution. Still, RNA analysis by NMR suffers from spectral overlap and line broadening, both of which worsen for larger RNAs. Incorporation of stable isotope labels into RNA has provided several solutions to these challenges. In this review, we summarize the benefits and limitations of various methods used to obtain isotope-labeled RNA building blocks and how they are used to prepare isotope-labeled RNA for NMR structure and dynamics studies.

## 1. Introduction

**Figure 1:**
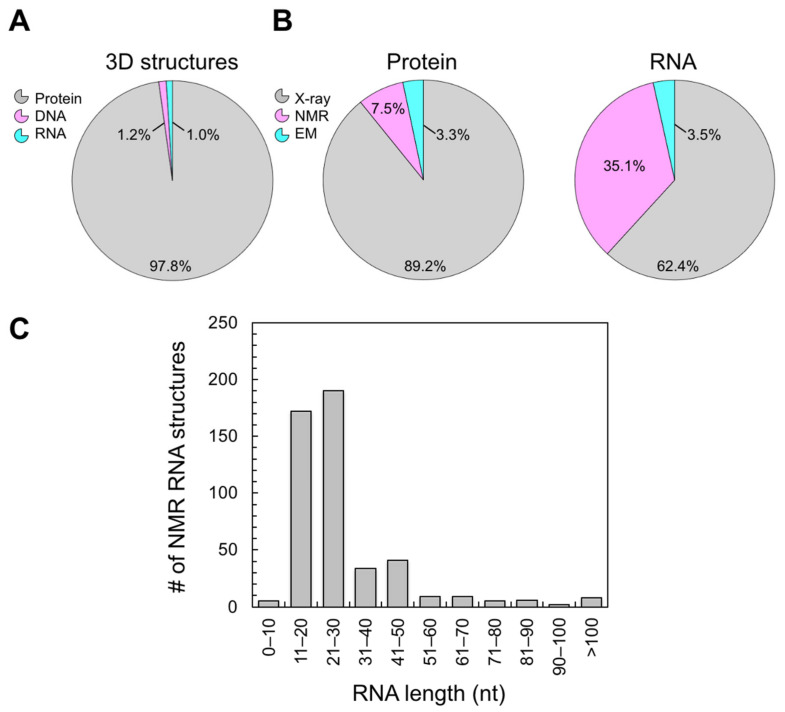
Structural biology statistics. The distribution of high-resolution 3D structures deposited in the PDB/NDB separated by (**A**) macromolecule type; and (**B**) protein and RNA structures separated by structure determination technique. (**C**) A histogram of RNA NMR structures in the NDB sorted by RNA length. Statistics were accessed in July 2021.

RNA is a dynamic macromolecule that has many biological functions, including gene regulation [1,2,3,4,5], catalysis [6,7,8], structural organization [9,10], and viral replication [11,12]. Almost without exception, RNA’s intricate three-dimensional (3D) structure and conformational plasticity are required to carry out these functions [13,14]. A robust understanding of RNA function therefore requires high-resolution structure and dynamics data. Unfortunately, there is a scarcity of RNA 3D structures as compared to those of proteins. There are only ~1500 RNA structures in the Nucleic Acid Database (NDB), whereas more than 150,000 protein structures are in the Protein Database (PDB) (Figure 1A). This observation can, in part, be explained by RNA’s dynamic nature, which can impede crystallization and complicate cryo-electron microscopy (cryo-EM) data analysis. While RNA’s conformational heterogeneity hinders X-ray crystallography and cryo-EM analysis, it is compatible with solution nuclear magnetic resonance (NMR) spectroscopy. Recent advances in NMR are even capable of probing low populated states that would otherwise be undetectable by traditional biophysical methods [15,16,17]. 

NMR is a competitive RNA structure determination technique that accounts for 35% of the structures in the NDB, as compared to the 8% of protein structures in the PDB (Figure 1B). However, obstacles remain. Specifically, NMR analysis of RNA suffers from spectral crowding and broad linewidths [18,19,20,21,22,23,24]. The former is a result of the limited chemical diversity of the RNA building blocks adenosine (A), guanosine (G), cytidine (C), and uridine (U), and the narrow chemical shift dispersion of ribose protons other than H1′ (i.e., H2′, H3′, H4′, H5′, and H5″). Both conditions worsen in larger RNAs and limit our understanding of their structures and functions. In fact, only 23 RNA structures >60 nucleotides (nt) have been solved by solution NMR (some requiring additional methodologies, e.g., cryo-EM) (Figure 1C). Advancements in stable isotope (e.g., ^2^H, ^13^C, ^15^N, and ^19^F) labeling of RNA have revolutionized the analysis of RNA by NMR and opened the door to functional understanding.

The main approaches to obtaining isotope-labeled RNA are enzymatic or chemical syntheses. For the enzymatic approach, almost all methods are based on DNA template-directed T7 RNA polymerase (RNAP)-based in vitro transcription (IVT) using labeled ribonucleoside 5′-triphosphates (rNTPs) [22,23,25,26,27,28,29,30,31]. The alternative method is chemical solid-phase synthesis (SPS) using RNA phosphoramidites (amidites) [32,33,34,35,36]. Both approaches can use unlabeled and isotope-labeled building blocks (rNTPs and amidites) to generate versatile RNA labeling patterns. The four strategies to obtain such building blocks for enzymatic RNA synthesis are: (1) purchase commercially available isotope-labeled rNTPs; (2) use simple organisms to incorporate isotope-labeled precursors into their rNTPs; (3) complete de novo biosynthesis of rNTPs; (4) utilize a hybrid chemo-enzymatic approach that combines chemical syntheses of ribose and nucleobases and their enzymatic coupling to prepare rNTPs. 

In this review, we detail the benefits and limitations of these four methods (Section 2). With labeled building blocks in-hand, we discuss the various ways of using them to make isotope-labeled RNA for NMR structure and dynamics studies (Section 3). Finally, we analyze isotope labeling in the context of RNA structural biology and comment on where we think the field is headed (Section 4).

## 2. Stable Isotope Labeling of RNA Building Blocks

Throughout this review, we follow the IUPAC/IUB guidelines for RNA atom numbering [37,38]. When describing RNA labeling, we use four categories: uniform, nucleotide-specific, atom-specific, and position-specific. Uniform labeling is when every atom of a certain type (e.g., ^2^H, ^13^C, ^15^N, or both of the latter) is enriched; nucleotide-specific labeling is when every nucleotide of a certain type (e.g., all uridines) is enriched; atom-specific labeling is when every atom of a certain type (e.g., uridine C6) is enriched; and position-specific labeling is when an individual nucleotide (e.g., uridine 7) is labeled. In the latter case, the type of label that is incorporated site-specifically can be uniformly or atom-specifically-labeled. Thus, these labeling categories are not mutually exclusive. 

### 2.1. Commercial Isotopes Sources

The simplest approach to obtaining isotope-labeled rNTPs and amidites is to purchase the desired ^2^H, ^13^C, and/or ^15^N isotope labels from a commercial source. As of July 2021, Cambridge Isotope Laboratories (CIL), Sigma-Aldrich, Cassia LLC, Silantes, and INNotope are the major suppliers of isotope-labeled rNTPs, whereas Silantes and INNotope are the only suppliers of isotope-labeled RNA amidites. Unfortunately, these products can be prohibitively expensive. Uniformly-labeled ^2^H, ^13^C, ^15^N, ^2^H/^15^N, and ^13^C/^15^N-labeled and atom-specifically ^2^H, ^13^C, ^2^H/^13^C-labeled rNTPs are available for $800–5600 per 100 μmol or 50 mg (Table 1). Additionally, uniformly ^13^C, ^15^N, and ^13^C/^15^N-labeled and atom-specifically ^2^H, ^13^C, ^2^H/^13^C, and ^15^N-labeled amidites are available for $900–6600 per 50 mg (Table 1). For reference, IVT (20 mL) typically requires 250–1000 μL (100 μmol stock) per rNTP and yields 0.2–2.0 mM in 300 μL of RNA (Section 3.2). SPS (1 μmol) generally requires 10–20 mg (0.1 M stock) per amidite coupling and yields 0.2–0.6 mM in 300 μL of RNA (Section 3.1). 

These considerations underscore a sobering fact: the approximate cost per NMR sample when using commercial building blocks often exceeds $1000. Since it takes multiple samples for complete RNA resonance assignment, total costs of robust RNA analysis by NMR can easily reach $10,000 based on isotope labeling costs alone. For example, a recently determined structure of the 43 nt SAM/SAH-binding riboswitch [39,40] required 7 uniform and nucleotide-specifically-labeled samples by IVT and 13 atom- and position-specifically-labeled samples for SPS. This financial burden partly explains the slow rate of RNA structure depositions (Figure 1A). It is therefore crucial to reduce the costs of obtaining isotope-labeled RNA and to expand the accessibility of NMR analysis of RNA.

### 2.2. Biomass Labeling

Biomass labeling incorporates isotope-labeled building blocks into simple organisms’ RNA. This labeling approach was established by the Pardi [22] and Williamson [23] research groups. In general, their method includes growing organisms on ^13^C and/or ^15^N source(s), harvesting cells and extracting RNA, hydrolyzing RNA to rNMPs, and converting those to rNTPs. Triphosphate conversion can be achieved by chemical [41] or enzymatic [42] means, depending on the expertise and resources available. The latter method is usually superior to the chemical approach, yielding rNTPs of >95% purity [22,23,41,42,43]. Although biomass methods permit new and commercially unavailable rNTP labeling patterns, the overall cost advantage is minimal, and the purification steps are laborious. Nevertheless, many research groups have utilized biomass labeling to prepare RNA for NMR analysis [22,23,41,42,43,44,45].

#### 2.2.1. Biomass Uniform Labeling

**Figure 2:**
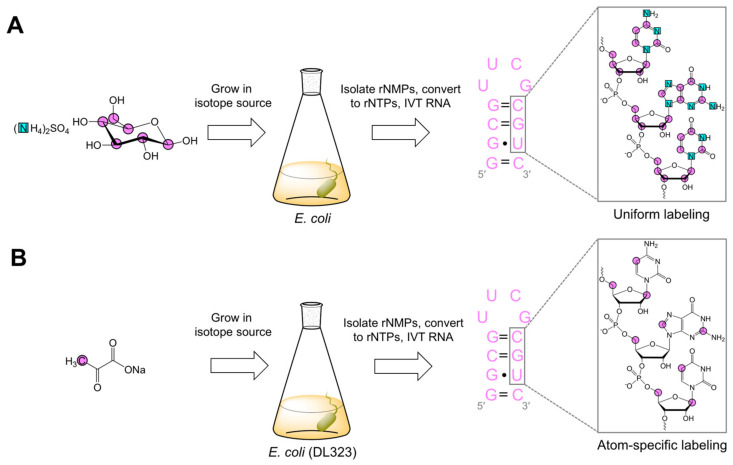
Schematic representations of biomass labeling adapted from the Pardi [22] and Williamson [23] research groups, and our previous work [44]. (**A**) *E. coli* grown in ^13^C-glucose and ^15^N-ammonium sulfate yields uniformly ^13^C/^15^N-labeled rNMPs, which can be phosphorylated, forming rNTPs, and then used in IVT to make uniformly (or nucleotide-specifically) labeled RNA. (**B**) *E. coli* (DL323) grown in [3-^13^C]-pyruvate affords rNTPs, which can then make atom-specifically-labeled RNA. Magenta circles and cyan squares represent ^13^C and ^15^N, respectively. Additional details can be found in the original works [22,23,44].

Uniform ^13^C labeling was first achieved by growing *E. coli* [22,23], *M. methylotrophus* [22], or *M. extorquens* [23] with either ^13^C-glucose or ^13^C-methanol. For uniform ^13^C/^15^N labeling, *E. coli* was grown with ^13^C-glucose and ^15^N-ammonium sulfate (Figure 2A) [22,23]. The use of *M. methylotrophus* and *M. extorquens* gained popularity due to their compatibility with the more cost-effective ^13^C-methanol [22]. Nevertheless, these organisms have significantly lower rNTP contents and more difficult growth conditions as compared to *E. coli* [22]. The rNTPs obtained from this method were used in IVT to make uniformly and nucleotide-specifically-labeled RNAs for multi-dimensional NMR experiments [22,23,43,46]. These new experiments greatly simplified resonance assignment strategies and the structure determination of small (<30 nts) RNAs. However, alternative labeling strategies were needed to overcome spectral crowding in larger RNAs. 

#### 2.2.2. Biomass Atom-Specific Labeling

Hoffman and Holland modified the biomass method to make atom-specifically-labeled rNTPs [47]. In their approach, *E. coli* were grown with different ^13^C-sodium acetate sources to make various ^13^C-labeled rNTPs. For example, [2-^13^C]-sodium acetate labeled purine (A and G) C2, C5, and C8 (>95%); pyrimidine (U and C) C5 and C6 (>90%); and ribose C1′, C4′, and C5′ (~90%). In addition, they made [1-^13^C]-sodium acetate labeled purine C4 and C6 (>90%), pyrimidine C2 and C4 (>95%), and ribose C3′ (~75%) [47]. Among others, Hoogstraten and co-workers employed a similar methodology wherein *E. coli* strains deficient in enzymes involved in the tricarboxylic acid cycle (DL323) [45] or the oxidative pentose phosphate pathway (K10-1516) [48] were grown with various ^13^C-glycerol sources [49]. While most labeling patterns had yields <50%, [2′,4′-^13^C_2_]-AMP was created with an 80% yield in K10-1516 cells grown with [2-^13^C]-glycerol. Importantly, Hoogstraten and co-workers measured longitudinal (R_1_) and rotating-frame (R_1ρ_) relaxation rates in [2′,4′-^13^C_2_]-AMP and uniformly ^13^C-labeled AMP to demonstrate that ^13^C–^13^C dipolar couplings lead to over-estimated relaxation rates [49].

Our research group used a similar method but with a different isotope source [44]. Purine C2 and C8 (~95%), pyrimidine C5 (~98%), and ribose C1′ (42%) and C5′ (95%) were labeled in DL323 cells fed with [3-^13^C]-pyruvate (Figure 2B). To demonstrate the utility of these labeling patterns, we used our atom-specifically-labeled rNTPs to make a 27 nt RNA via IVT for NMR analysis. In agreement with previous work, R_1_ rate measurements showed a discrepancy between uniformly and atom-specifically-labeled samples for pyrimidine C5 and ribose C1′ and C5′ [44,49].

### 2.3. Ribonucleotide De Novo Biosynthesis

A ribonucleotide de novo biosynthesis uses enzymes from the pentose phosphate and nucleotide salvage biosynthetic pathways (Table 2), various cofactor regeneration systems, and isotope-labeled precursor compounds to synthesize purine [27] and pyrimidine [28] rNTPs in a one-pot enzymatic reaction. The benefits of this route include reduced reaction time and increased product yield and specificity, compared with traditional chemical synthesis [50,51,52,53,54]. Moreover, this approach produces cost-effective uniformly ^13^C/^15^N-labeled and atom-specifically-labeled rNTPs. On the other hand, laborious protein cloning is involved, but the pay offs in preparation and purification more than make up for this initial outlay. Still, de novo labeling to prepare RNA for NMR studies has been used with some success [27,28].

#### 2.3.1. Purine De Novo Biosynthesis

**Figure 3:**
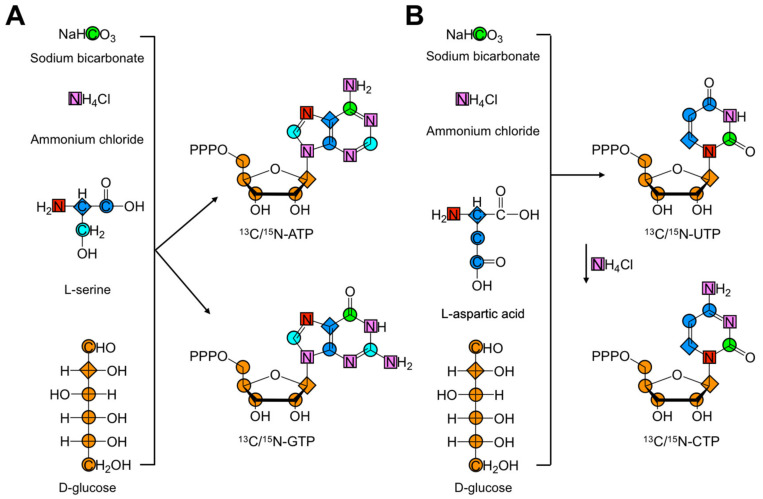
De novo biosynthesis of rNTPs adapted from Williamson and co-workers [27,28]. The four isotope-labeled precursors needed for the de novo biosynthesis of uniformly ^13^C/^15^N-labeled (**A**) purine and (**B**) pyrimidine rNTPs are shown, and the heteroatoms are color coded to match their corresponding labeling positions. As shown in (**B**), UTP is converted to CTP in one additional step. For consecutive carbon atoms that are incorporated concomitantly, the first position is diamond shaped to indicate the direction of incorporation. All colored shapes represent either a ^13^C or a ^15^N atom. The isotope label of the C1 of glucose can complicate the label of C6 in the purine nucleobase. Care is usually taken to prevent dilution. Additional details can be found in the original works [27,28].

Williamson and co-workers were the first to describe the de novo biosynthesis of isotope-labeled purine rNTPs [27]. Their approach used enzymes from the pentose phosphate pathway to convert glucose to 5-phospho-D-ribosyl-α-1-pyrophosphate (PRPP) and enter a linear cascade of reactions to assemble the purine ring and produce inosine monophosphate (IMP), a precursor for both ATP and GTP. The de novo biosynthesis of ATP and GTP also required NAD(P)H and rNTP regeneration systems, folate, aspartate, and glutamine. Isotope-labeled precursor compounds ^13^C-D-glucose, ^13^C-sodium bicarbonate (NaH^13^CO_3_), ^15^N-ammonium chloride (^15^NH_4_Cl), and ^13^C/^15^N-L-serine enabled atom-specific labeling. As shown in Figure 3A, purine labeling is provided by these precursor compounds. Specifically, N1, N2, N3, N6, and N9 are derived from ^15^NH_4_Cl. Similarly, ^13^C/^15^N-L-serine labels C2, C4, C5, C8, and N7, and NaH^13^CO labels C6. Finally, ^13^C-D-glucose provides the label for the ribose carbons (C1′, C2′, C3′, C4′, and C5′) and C6. 

While this methodology is very powerful, it also comes with a number of drawbacks. The use of labeled glucose can limit the potential labeling patterns if both ribose and nucleobase labels are desired. For example, production of CO_2_ via decarboxylation of 6-phosphogluconate to ribulose-5-phosphate during PRPP production links the isotope label of the C1 of glucose to C6 in the purine nucleobase. If ribose labeling is not required, and only purine C6 labeling is needed, then PRPP must be made directly from unlabeled ribose. Similarly, care must be taken to prevent isotopic dilution from atmospheric CO_2_, if both ^13^C-ribose and ^13^C6-purine labeling are required. Additionally, the C2 and C8 positions are labeled together or not at all. Finally, C6 and ribose labeling are limited by commercial sources of D-glucose.

In summary, a total of 28 biosynthetic enzymes (Table 2) were used for the efficient one-pot synthesis of ATP and GTP over two days, producing yields of up to 66% [27]. Specifically, 23 enzymes were used to synthesize [2,8-^13^C_2_]-ATP in a 57% yield and 26, 24, and 27 enzymes helped synthesize uniformly ^13^C, ^15^N, ^13^C/^15^N-labeled GTP in 66, 24, and 42% yields, respectively [27]. Their atom-specific rNTPs were used in IVT to make a 30 nt RNA for NMR analysis. Their labeling patterns helped identify specific nucleobase interactions and greatly reduced spectral crowding.

#### 2.3.2. Pyrimidine De Novo Biosynthesis

Extending previous work, Williamson and co-workers developed the first de novo biosynthesis of isotopically labeled pyrimidines [28]. In contrast to purine synthesis, where the nucleobase was constructed step-by-step on the ribose, the nucleobase was directly and enzymatically coupled to the ribose to synthesize pyrimidines. Rather than directly coupling uracil, orotidine 5′-monophosphate (OMP) was produced and then converted to UTP. In a final step, UTP was converted to CTP with CTP synthetase (CTPS) (EC 6.3.4.2, PDB ID: 6NUI) and NH_4_Cl [55]. This method still relied on enzymes from the pentose phosphate and nucleotide salvage biosynthetic pathways, albeit with two enzymes cloned from species other than *E. coli*: the carbamoyl-phosphate synthase-like carbamate kinase enzyme (cpkA) was cloned from the thermophile *Pyrococcus furiosus* (EC 6.3.5.5, PDB ID: 1E19), and dihydro-orotate dehydrogenase A (pydA) from *Lactoccocus lactis* (EC 1.3.5.2, PDB ID: 2DOR). The de novo biosynthesis of UTP and CTP also required ATP and NADPH regeneration systems and isotope-labeled precursor compounds ^13^C-D-glucose, NaH^13^CO_3_, ^15^NH_4_Cl, and ^13^C/^15^N-L-aspartate. Figure 3B shows the sources of pyrimidine rNTP labels. Specifically, C4, C5, C6, and N1 were derived from ^13^C/^15^N-L-aspartate; N3 and CTP N4 were delivered by ^15^NH_4_Cl; and C2 came from NaH^13^CO_3_. All ribose carbons were provided by ^13^C-D-glucose. Again, this labeling methodology has a number of drawbacks. The use of labeled glucose can limit the potential labeling patterns if both ribose and nucleobase labels are desired. For example, production of CO_2_ via decarboxylation of 6-phosphogluconate to ribulose-5-phosphate during PRPP production, and of OMP to UMP, links the isotope label of the C1 of glucose and the C1 of aspartate to C2 in the pyrimidine nucleobase. If ribose labeling is not needed and only pyrimidine C2 labeling is wanted, then PRPP must again be made directly from unlabeled ribose. Similarly, care must be taken to prevent isotopic dilution from solvent and atmospheric CO_2_, if both ^13^C-ribose and ^13^C2-pyrimidine labeling are required. Alternatively, commercially available ^13^C1-aspartate can be used to label C2 without C4, C5, and C6 labeling. 

To summarize, a total of 16 biosynthetic enzymes were used for the efficient one-pot synthesis of UTP (and CTP) over 3–4 days with yields of up to 45% [28]. Specifically, 15 enzymes were used to synthesize atom-specific [1′,6-^13^C_2_]-UTP with a 25% yield; and batches of 15, 16, and 16 enzymes helped synthesize uniformly ^13^C, ^13^C/^15^N, ^15^N-[5,3′,4′,5′,5″-^2^H_5_]-labeled UTP with 40, 45, and 30% yields, respectively [28]. Additionally, CTPS converted uniformly ^15^N-[5,3′,4′,5′,5″-^2^H_5_]-UTP to its CTP counterpart with a 48% yield. The utility of their UTP and CTP labels was demonstrated for the same 30 nt RNA model system. This labeling scheme reduced spectral crowding even more than before, owing to ribose deuteration. 

### 2.4. Chemo-Enzymatic Labeling

Chemo-enzymatic labeling is a hybrid approach that we developed, taking inspiration from the Tinoco [29] and Williamson [27,28] research groups. In brief, this method uses enzymes from the nucleotide salvage biosynthetic pathways and cofactor regeneration systems to couple a nucleobase and ribose, followed by subsequent phosphorylation to the rNTP in a one-pot enzymatic reaction [30,31,56]. Moreover, the nucleobase and ribose building blocks can be unlabeled, isotope-labeled, chemically synthesized, or commercially available, permitting a diverse set of labeling patterns. In collaboration with the Kreutz research group, we have prepared rNTPs with a variety of commercially unavailable labeled nucleobases at reduced costs [30,31]. This approach has many advantages over previously reported de novo [27,28] and chemical [50,51,52,53,54] synthesis methods, including fewer enzymes, fewer synthetic steps, and greater yields. 

We described the first chemo-enzymatic synthesis of [1′,5′,6-^13^C_3_-1,3-^15^N_2_]-UTP and -CTP in 90 and 95% yields, respectively, by coupling [6-^13^C_2_-1,3-^15^N_2_]-uracil to [1,5-^13^C_2_]-ribose [30]. These atom-specifically-labeled rNTPs were then used in IVT to make a variety of RNAs ranging in size from 27 to 155 nts. This RNA labeling scheme significantly reduced spectral crowding, increased signal-to-noise ratios, facilitated direct carbon detection experiments, and eliminated ^13^C-^13^C scalar and dipolar couplings. The latter benefit alleviated systematic overestimations in C1′ R_1_ rates that were observed in uniformly ^13^C/^15^N-labeled RNA, in agreement with previous reports [30,44,49]. Extending this work, we also detailed the first chemo-enzymatic synthesis of various atom-specifically-labeled ATPs and GTPs by combining [8-^13^C]-adenine or [8-^13^C]-guanine with either [1-^13^C], or [2-^13^C], or [1,5-^13^C_2_]-ribose to yield the desired rNTPs in 70–95% yields [31]. These atom-specifically-labeled ATPs and GTPs were then used (along with the previously described CTP and UTP) in IVT to make a variety of RNAs ranging in size from 27 to 59 nts. This labeling pattern offered substantial sensitivity improvements and was used to develop a novel resonance assignment strategy [57]. Additional synthetic details, including a list of enzymes used, can be found in the original works [30,31,56], and the method has also been used in recent studies by our group [58,59]. This topic will also be the focus of an upcoming review.

It is worth noting that there are other approaches to enzymatically coupling nucleobase and ribose sources. Indeed, the synthetic method of Serianni and co-workers begins with the chemical synthesis of inosine, followed by its phosphorylation by purine nucleoside phosphorylase (PNPase) (EC 2.4.2.1) to give α-D-ribofuranosyl 1-phosphate sodium salt (αR1P) [60]. The αR1P intermediate is then glycosylated enzymatically to the ribonucleoside which can easily be converted to the desired rNTP. 

### 2.5. RNA Phosphoramidite Labeling

Thus far, all discussions of isotope-labeled building blocks (Section 2.2, Section 2.3 and Section 2.4) have focused on rNTPs for use in IVT of RNA. However, RNA can also be prepared by chemical SPS with unlabeled or isotope-labeled amidites. Nearly all amidite synthetic schemes have nucleoside intermediates. Thus, any method to incorporate isotope labels into nucleobases, nucleosides, or rNTPs (Section 2.2, Section 2.3 and Section 2.4) can be converted into amidites. Indeed, in collaboration with the Kreutz group, we used the chemo-enzymatic method to make [1′,8-^13^C_2_]-AMP which was then dephosphorylated to adenosine and used as the entry point for amidite synthesis [61]. Several groups have also developed strategies to obtain ^13^C/^15^N-labeled amidites. Initial efforts were developed by the Pitsch [62] and Jones [63,64,65,66] research groups. More recently, the Micura [67,68] and Kreutz [17,61,69,70,71] research groups have dramatically improved the efficiency and scalability of amidite synthesis for NMR analysis. These methods were recently reviewed [36]. 

## 3. RNA Preparation Methods

With isotope-labeled building blocks in-hand, we can now discuss how they are used to prepare isotope-labeled RNA for NMR analysis. In general, amidites are used in chemical SPS to make small-to-medium sized RNAs (20–40 nts) with atom- and position-specific labeling. However, recent work from the Kreutz research group extended this length to ~80 nts [69]. All other in vitro methods of RNA preparation require T7 RNAP based IVT and use rNTPs. IVT is the most widely used method to prepare medium sized RNAs (~20–100 nts) but has major NMR limitations due to spectral overlap. Nevertheless, large RNAs can still be profitably analyzed if made from atom-specifically-labeled rNTPs [30,31,61,72,73,74,75]. The remaining RNA preparation methods rely on segmental labeling of large RNAs (>100 nts) from smaller fragments or enzymatic incorporation of position-specific isotope labels into the RNA.

### 3.1. Chemical Solid-Phase RNA Synthesis

**Figure 4:**
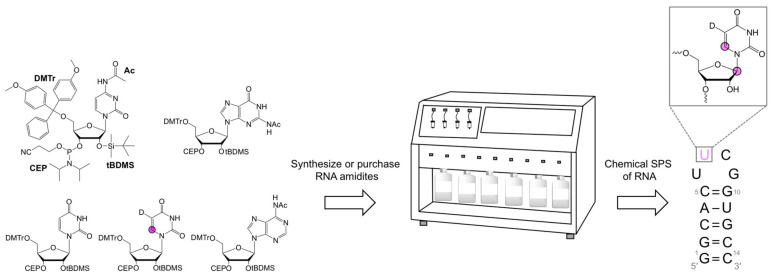
Chemical solid-phase synthesis of position-specifically-labeled RNA. Amidite building blocks (unlabeled and isotope-labeled) are used in an automated synthesizer to make pre-programmed RNA sequences with position-specific labels. Magenta nucleotides harbor stable isotope labels. Magenta circles and D represent ^13^C and ^2^H atoms, respectively. This example shows a [6-^13^C-5-^2^H]-UTP-labeled RNA at nucleotide U7.

Originally developed for DNA synthesis by Beaucage and Caruthers [76], the amidite method has since been adapted to RNA [32,33,34,35,36]. This approach has been further adapted to incorporate isotope-labeled amidites [36]. SPS is carried out in an automated synthesizer, requires amidite building blocks, and occurs in four steps (Figure 4). First, the 4,4′-dimethoxytrityl (DMTr) protecting group at the 5′-hydroxyl (OH) of the solid-support bound 3′-nucleoside is removed. Second, the deprotected 5′-OH then attacks the activated amidite to couple the two nucleosides. Synthesis continues to grow the RNA molecule 3′-to-5′ by repeating the first two steps following oxidation of the phosphite-triester to the phosphotriester and subsequent deprotection. Cleavage from the solid-support terminates the cycle.

SPS efficiency depends on the protecting group (PG) choice. RNA amidites are 5′-O-DMTr-protected, and the nucleobase exocyclic amino groups are protected with acetyl (Ac), phenoxyacetyl (Pac), benzoyl (Bz), or isobutyryl (iBu) groups. The choice of the various 2′-OH PGs requires careful deliberation. These PGs can be classified as acid- [77], photo- [78], and fluoride-labile [33,79,80]. While RNAs have been synthesized with a variety of 2′-OH PGs, only [(triisopropylsilyl)oxy]methyl (TOM) [79], *tert*-butyldimethylsilyl (tBDMS) [33], and 2′-cyanoethoxymethyl (CEM) [80] amidites have been widely used in NMR studies. Even though the CEM is the only PG that enables synthesis of RNAs >60 nts, it is commercially unavailable, which requires researchers to synthesize unlabeled and labeled CEM amidites in-house [69]. On the other hand, unlabeled TOM and tBDMS amidites and isotope-labeled tBDMS amidites are commercially available (Table 1) and therefore more accessible to a larger group of researchers. In practice, chemical SPS is rarely employed to make RNAs >60 nt for NMR analysis, and therefore TOM and tBDMS amidites are mainly used. For example, the Kreutz research group synthesized [6-^13^C]- and [6-^13^C-5-^2^H-]-uridine and -cytidine, and [2,8-^13^C_2_]- and [8-^13^C]-adenosine and -guanosine TOM and tBDMS amidites for use in ^1^H- [70] and ^13^C-relaxation dispersion [17,71] NMR experiments to study conformational dynamics of RNAs up to 53 nts. 

### 3.2. T7 RNA Polymerase-Based In Vitro Transcription

IVT with DNA-dependent RNA polymerases from bacteriophage SP6, T3, or T7 (EC 2.7.7.6) is a widely used enzymatic method for RNA synthesis (Table 3) [25,26,81,82,83]. T7 RNAP IVT is undoubtedly the standard approach for making RNAs for NMR analysis. In practice, IVT is performed with chemically synthesized single-stranded or double-stranded DNA templates comprising one of two T7 RNAP promoter sequences (class II φ2.5 or class III φ6.5) [26,83,84]. While this approach overcomes the size restrictions of chemical SPS, it has limitations of its own. First, the widely used class III promoter is GTP-initiated and requires 5′-GG for efficient initiation [83]. Second, repeated failed transcription initiation results in 5′-end heterogeneity [85,86]. Third, T7 RNAP often adds additional non-templated rNTPs to the 3′-end of the nascent RNA [26,87]. Lastly, T7 RNAP is not immediately amenable to position-specific labeling of RNA, though some exceptions may arise. Fortunately, the 3′- and 5′-end heterogeneities are dramatically reduced by incorporating ribozyme sequences in the template in cis and trans [88,89], by chemically incorporating 2′-O-CH_3_ rNTPs at the 3′-end [90], or by judicious choice of 5′ sequences that minimize 5′-end heterogeneity (Table 4). In addition to template modification, the efficiency of T7 RNAP IVT with nucleotides bearing 2′-O-F, 2′-O-NH_2_, or 2′-O-CH_3_ modified ribose is enhanced by introducing Y639F and H784A mutations [91,92,93,94]. Although the addition of non-templated rNTPs remains a challenge to T7 RNAP IVT, Roy and co-workers found no detectable 3′-end products when transcribing RNA of various sizes at higher temperature [95].

Despite its limitations, T7 RNAP IVT is an extremely versatile method and is compatible with unlabeled and isotope-labeled rNTPs. The latter can either be purchased (Section 2.1) or prepared in-house by biomass, de novo biosynthesis, or chemo-enzymatic methods (Section 2.2, Section 2.3 and Section 2.4). As such, this method can yield uniformly, nucleotide-specifically, and atom-specifically-labeled RNA. In the latter case, the preparation of deuterated RNA greatly simplifies NMR spectra and reduces dipolar relaxation. Deuteration of the H1′, H2′, H3′, H4′, and H5′/5′′ positions in the ribose and H5 position in C/U nucleobases can be achieved with commercially available rNTPs (Table 1). On the other hand, atom-specific deuteration of purine H8 can be accomplished by heating the purine nucleobase to 55 °C in D_2_O [21,96] and helps distinguish adenine H8 from guanine H8 and adenine H2 [74]. These methods have been extended by the Summers research group [72,73,74,75] to study very large RNAs. Similarly, uracil H5 is easily deuterated using D_2_O and triethylamine [70] and then readily converted to [5-^2^H]-UTP and [5-^2^H]-CTP enzymatically [57]. This specific deuteration eliminates competing relaxation pathways among dipolar coupled protons such as H5–H6, leading to sharp linewidths, increased signal-to-noise, and reduced chemical shift overlap in NMR spectra [19]. Using these strategies, perdeuteratated, fully protonated, and atom-specifically deuterated samples were prepared by IVT (and sometimes segmental labeling schemes, see Section 3.3.2) to overcome the ambiguous assignment problems of large RNAs and determine their structures [72,73,74,75].

**Table 4:** Sequences requirements for maximum RNA 5′-end homogeneity in T7 RNA polymerase in vitro transcription.

5′-End Sequences ^a^	5′-End Heterogeneity (<1%)
GGG	No
GAG	No
GCG	No
GUG	Yes
GGA	Yes
GAA	Yes
GCA ^b^	Yes
GUA	Yes
GGC	No
GAC	Yes
GCC	No
GUC	Yes
GGU	No
GAU	Yes
GCU	Yes
GUU	Yes
AGG	No
AAG	Yes
ACG	Yes
AUG ^b^	Yes
AGA	No
AAA	No
ACA	No
AUA	No
AGC	Yes
AAC	No
ACC	No
AUC	No
AGU ^b^	Yes
AAU	No
ACU	Yes
AUU	Yes

^a^ Sequences synthesized from either wild type or mutant T7 (p266L) RNA polymerase [97]. ^b^ Sequences synthesized from mutant p266L T7 only. Sequences starting with a G were synthesized with the T7 RNA polymerase bearing a class III promoter. T7 RNA polymerase with a class III ϕ2.5 promoter was used for sequences starting with an A. All but three of the sequences, as reported by Legault and co-workers, were synthesized with either the wild type or mutant T7 [98].

### 3.3. Enzymatic Ligation

One approach that enables position-specific and segmental isotope labeling is the ligation of two RNA molecules by T4 DNA (EC 6.5.1.1) or RNA ligase (EC 6.5.1.3) (Table 3). These ligating enzymes have also been combined with self-cleaving ribozymes to segmentally label RNA. In these methods, multiple fragments of RNA are ligated to produce a larger isotope-labeled RNA that can be studied by NMR. Depending on the RNA sequence under investigation, researchers can devise unique labeling patterns to incorporate position-specific labels and greatly reduce spectral overlap and NMR analysis.

#### 3.3.1. T4 DNA and RNA Ligation 

**Figure 5:**
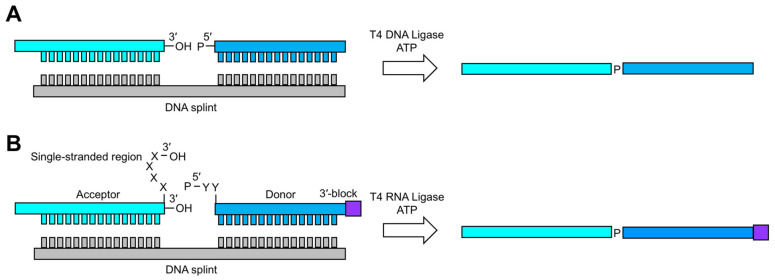
Enzymatic RNA ligation [19,99]. DNA splinted ligation schemes are shown using (**A**) T4 DNA ligase and (**B**) RNA ligase. Shown in **B** are the optimized 3′-end linker and 3′-end block of the donor and acceptor fragments, respectively. Sequence requirements for T4 RNA Ligase are also shown. Additional details can be found in earlier works [19,99].

The standard method for RNA ligation uses T4 DNA ligase (EC 6.5.1.1) [99]. In the presence of ATP, this enzyme recognizes a nicked double-stranded substrate and joins a 5′-monophosphate (P) RNA (donor) with a 3′-OH RNA (acceptor) (Figure 5A). The donor and acceptor RNA fragments can either be prepared by chemical SPS or IVT. In the former case, the 5′-P can be added during or after donor RNA synthesis using T4 polynucleotide kinase (T4 PNK) (EC 2.7.1.78) and ATP. In the latter case, a donor RNA can be initiated with GMP or a 5′-XpG-3′ dinucleotide. Alternatively, transcribed RNAs can be dephosphorylated with recombinant shrimp alkaline phosphatase (rSAP) (EC 3.1.3.1) and then phosphorylated with T4 PNK [100]. The 3′- and 5′-end heterogeneities dramatically reduce ligation efficiency, and therefore great care must be taken to purify the RNA of interest [99]. The two main advantages of T4 DNA ligation are that undesired side products (e.g., circularization and oligomerization) are minimized and enzymatic activity is independent of ligation junction sequence. A major disadvantage of the methodology is that T4 DNA ligase requires large quantities of RNA and is relatively inefficient at joining RNA strands [99].

An alternative method for RNA ligation is using T4 RNA ligase (EC 6.5.1.3). Like its DNA counterpart, RNA ligase requires a 5′-P donor, a 3′-OH acceptor, and ATP (Figure 5B) [101]. However, RNA ligase requires single-stranded ligation junctions, complicating the use of cDNA as a template. To overcome this limitation, Bain and Switzer designed a DNA splint that positioned the donor and acceptor in close proximity [102]. This designed single-stranded region is compatible with T4 RNA ligase and resulted in ligation efficiencies of 53%. Building on this work, Rader and co-workers optimized ligation efficiency to near completion in less than an hour [103]. To achieve this, they (1) protected the donor 3’-OH with a 5′-silyl-2’-acetoxy-ethyl orthoester (2’-ACE) group to minimize side products, (2) chemically incorporated the 5′-P to minimize 5′-end heterogeneities, and (3) designed an optimized linker at the ligation junction. 

#### 3.3.2. Segmental RNA Labeling

Another unique ligation strategy employs RNAse H and hammerhead (HH), Varkud satellite (VS), and hepatitis delta virus (HDV) self-cleaving ribozymes. This approach dramatically reduces 3′- and 5′-end heterogeneities and has therefore been embraced as a popular method to segmentally label large RNAs for NMR studies. Two such examples have come from the Puglisi [104] and Lukavsky [105] research groups. Their method was streamlined by the construction of a plasmid containing a T7 RNAP promoter, the 3′-fragment and its 3′-HH ribozyme in cis confirmation, and the RNA of interest. Despite the attractive design, the protocol took 12–14 days and only yielded 20–22 nmol RNA [104,105]. Nevertheless, this approach enabled simplified NMR structural analysis of 74 nt [105] and 77 nt [104] RNAs. 

**Figure 6:**
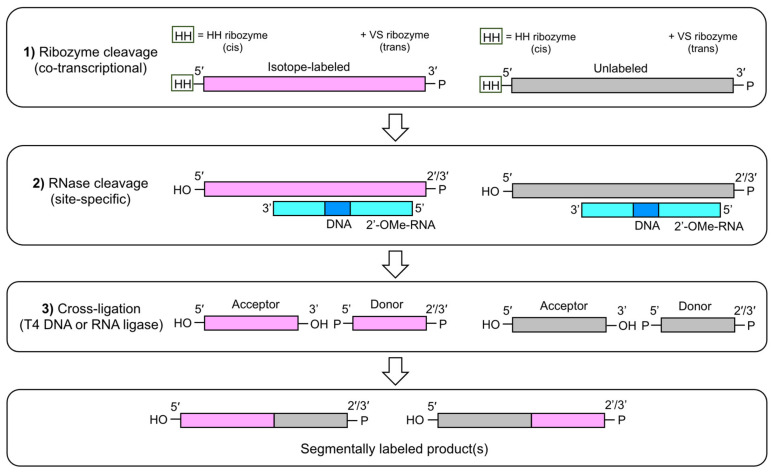
Segmental RNA labeling strategy adapted from Allain and co-workers [107]. Labeling is achieved via IVT of identical unlabeled and isotope-labeled and fragments along with HH (cis) and VS (trans) ribozymes which are cleaved co-transcriptionally (step 1). Then, site-specific RNase H cleavage is facilitated by a DNA/RNA chimera (step 2) following cross ligation reactions with T4 DNA or RNA ligases to yield segmentally labeled RNA (step 3). Additional details can be found in the original work [107].

Building on this work, Wijmenga and co-workers developed an efficient two-step ligation method to selectively label central positions of large RNAs [106]. The utility of these labeling patterns to simplify resonance assignment was showcased with a 61 nt viral RNA. Still, this method only yielded 15–30 nmol RNA and required 9–11 days [106]. Finally, Allain and co-workers developed an alternative approach for segmental labeling of RNA based on IVT of two full-length RNAs with identical sequence: one labeled and one unlabeled [107]. The RNAs were flanked at the 5′- and 3′-end by the HH and VS ribozymes, respectively. After ribozyme and RNase H cleavage steps, the acceptor and donor fragments were cross-ligated using T4 DNA or RNA ligase (Figure 6). The power of this method was demonstrated in a 72 nt non-coding RNA containing four stem-loops. Four NMR samples were made: each with only one of the four stem-loops isotope-labeled. This approach also provided ~10-fold better yield (90–260 nmol RNA) and required less time (5–7 days) than did previous methods [107]. 

### 3.4. Enzymatic Position-Specific RNA Labeling

The final two RNA preparation methods rely on enzymatic incorporation of position-specific isotope labels within RNA. These approaches hold promise for enabling site-specific NMR measurements of large RNAs, and therefore combine the benefits of both enzymatic and chemical SPS preparation methods. 

#### 3.4.1. Position-Selective Labeling of RNA (PLOR)

Wang and co-workers developed a powerful but laborious method to obtain position-specific isotope-labeled RNA [108,109,110]. This hybrid solid-liquid phase transcription technique uses an automated robotic platform known as position-selective labeling of RNA (PLOR) to prepare isotope-labeled RNAs in a three-step process that includes initiation, elongation, and termination. In PLOR, the DNA template is attached to beads and IVT is initiated by the addition of T7 RNAP and a mixture missing one of the four rNTPs, stalling RNA elongation. The beads are then washed to remove unincorporated rNTPs and elongation by T7 RNAP is resumed by the addition of a new mixture containing the previously omitted rNTP. Repetition of the elongation, stalling, and reinitiation steps enables synthesis of position-selective labeled RNA. The main drawbacks of this method are the inaccessibility of the automated synthesizer and the need for stoichiometric amounts of T7 RNAP and DNA template. Additionally, a stretch of identical nucleotides (e.g., UUU) cannot be individually labeled. Wang and co-workers showcased the utility of PLOR to aid NMR structural studies of a 71 nt RNA [108]. The power of PLOR for synthesizing site-specific and fluorescently labeled 140 nt viral RNA has also been reported [111]. Segmental labeling of the 71 nt RNA dramatically improved signal overlap and aided the unambiguous detection of multiple conformations of a single nucleotide in a position-specifically-labeled sample. While this method holds promise, the requisite equipment needed for segmental labeling with PLOR is not publicly available.

To facilitate the use of this technique for the synthesis of larger RNAs, Liu and co-workers recently characterized the sequence requirement for the initiation step which is crucial for an efficient synthesis of RNA at optimal yields [112]. From the study of 16 RNAs derived from the adenine (riboA) and thiamine pyrophosphate (riboTPP) riboswitches, it was observed that the first six nucleotides at the 5′-end were more crucial for the PLOR-based synthesis of RNA transcripts than the nucleotides following. In addition, and consistent with IVT where G nucleotides at the first and second position of the sequence are preferred by T7 RNA polymerase, the presence of a 5′-GGG sequence was optimal for all of the sequences studied [85,113,114,115]. Finally, initiation lengths between 11 and 19 nt were also reported to be crucial for the synthesis of RNA transcripts at optimal yield. Following this new discovery, the future of studying large (>60 nt), biologically relevant RNAs with PLOR looks promising, and addressing the hardware limitation of this method may help to make it more broadly adopted.

#### 3.4.2. Chemo-Enzymatic Position-Specific Labeling

**Figure 7:**
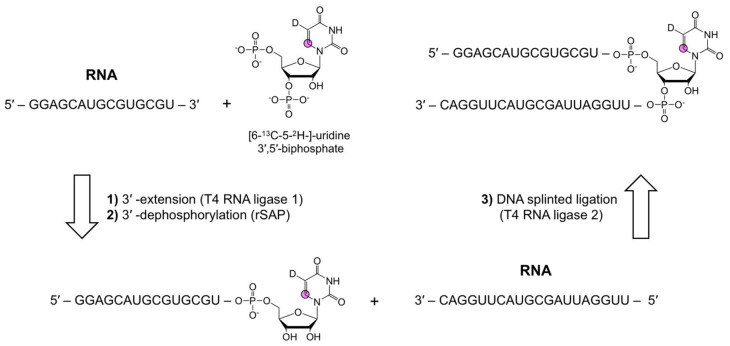
A position-specific RNA labeling strategy adapted from Schwalbe and co-workers [116]. Labeling is achieved via 3′-end extension with a modified 3′,5′-biphsophate nucleoside, followed by 3′-end dephosphorylation of the nucleoside by T4 RNA ligase 1 and rSAP. Lastly, DNA splinted ligation with T4 RNA ligase 1 combines the modified RNA with a 5′-phoshporylated donor RNA. Additional details can be found in the original work [116].

In an attempt to make position-specifically-labeled RNA more accessible, Schwalbe and co-workers developed a chemo-enzymatic synthetic approach. Therein, a single modified nucleoside-3′,5′-bisphosphate is incorporated to the 3′-end of an RNA fragment followed by DNA-splinted ligation to complete the desired internally labeled RNA sequence (Figure 7) [116]. This method was used to introduce photocaged, photoswitchable, and isotope-labeled ribonucleosides into RNAs of up to 392 nts. Furthermore, this method uses standard laboratory equipment and the commercially available enzymes T4 RNA ligase 1, rSAP, and T4 RNA ligase 2, making it readily accessible to most research groups. However, the relatively low yields of bis-phosphorylation (6–22%) and ligation (9–49%) reactions are a major drawback of this approach [116]. Improvements in these reaction steps would encourage the widespread adoption of this methodology. 

## 4. Conclusions

We have presented a detailed overview of the various methods for obtaining isotope-labeled rNTPs (Section 2) and how they are used to prepare isotope-labeled RNA for NMR structure and dynamics studies (Section 3). Despite these advances, there is still a tremendous bottleneck for complete RNA resonance assignment, which is a prerequisite for RNA structure determination. On the one hand, there are dramatic costs associated with NMR sample preparation. Many samples are needed for complete RNA resonance assignment, and even more are needed for RNA structure determination. In addition, extreme time investments are required to characterize RNAs. The work needed to determine the structure of a medium-sized RNA often spans an entire PhD or post-doc, if not more. Our example from Section 2.1 illustrates this point. The solution NMR-based structure determination of a 43 nt RNA required 20 NMR samples and 10 contributing authors [39,40]. The costs of materials for NMR sample preparation and the labor are prohibitively expensive for most research groups. Moreover, the aforementioned RNA is only of modest size; studying larger RNAs will involve greater financial and time commitments.

However, RNA structural biology is moving toward larger and larger RNAs, especially as cryo-EM gains in resolution and popularity [117,118]. Nevertheless, solution NMR studies, unlike X-ray crystallography and cryo-EM, attempt to replicate the appropriate physiological environments and temperatures, and are therefore more apt for investigating the structural dynamics (from picoseconds-to-seconds) of macromolecules. Certainly, the technical advances and explosion of new data from X-ray and/or cryo-EM structures portend exciting times for the use of solution NMR in integrative structural biology and drug discovery projects [73,118,119,120,121]. Still, the challenges associated with NMR studies of large systems must be met head-on. Attention must center on either (1) developing approaches that are capable of efficient position-specific or segmental labeling of large RNAs, (2) strategic “divide-and-conquer” designs of atom-specifically-labeled RNAs, or (3) reducing the cost of selective deuteration. In the first case, improving ligation efficiency would open the door for larger RNAs to be constructed from any variations of atom-specifically-labeled RNAs from IVT and/or position-specifically-labeled RNAs from SPS. Secondly, careful design of multiple small, functional, and folded core RNAs that represent larger RNAs would be powerful if used in combination with atom-specific labeling. Indeed, this “divide-and-conquer” strategy has been used successfully to study RNAs as large as 155 nts [73,74,122]. Lastly, NMR experiments with selectively deuterated rNTPs have been used with great success by the Summers research group to determine the structures of large RNAs [72,73,74,75]. However, the extreme prices of these rNTPs prevent their widespread use (Table 1). Taken together, analysis of large RNAs by solution NMR spectroscopy will always be a challenge. Such studies will involve trade-offs between costs and efficient labeling methods. Fortunately, the methodological developments described herein demonstrate a research community that has adapted to previous challenges and will continue to do so. 

## Figures and Tables

**Table 1:** Price of commercial isotope-labeled rNTPs and RNA amidites.

Building Block	Price ^a^ ($)	Supplier ^b^
**Uniformly ^2^H-labeled rNTPs**		
rNTP (N = A, C, G, or U)	1300	Silantes
**Selectively ^2^H-labeled rNTPs**		
[3′,4′,5′,5″-^2^H_4_]-X (X = ATP or GTP)	800	CIL
[2-^2^H]-ATP	1200	CIL
[5,1′,2′,3′,4′,5′,5″-^2^H_7_]-CTP	1800	CIL
[5,3′,4′,5′,5″-^2^H_5_]-Y- (CTP or UTP)	800	CIL
[5,6-^2^H_2_]-CTP	1800	CIL
[5,1′,2′,3′,4′,5′,5″-^2^H_7_]-UTP	1400	CIL
**Uniformly ^13^C-labeled rNTPs and Amidites**		
rNTP (N = A, C, G, or U)	1400	Silantes
N (N = A, C, G, or U Amidites)	6600	Silantes
**Selectively ^13^C-labeled rNTPs and Amidites**		
[8-^13^C]- X (X = ATP or GTP)	1400	Silantes
[8-^13^C]-A	900	Silantes
[2,8-^13^C_2_]-A	2700	Silantes
[8-^13^C]-G	1000	INNotope
**Selectively ^2^H/^13^C-labeled rNTPs and Amidites**		
[6-^13^C-5-^2^H]- Y (Y = CTP or UTP)	1600	Silantes
[6-^13^C-5-^2^H]-C or U	1000	INNotope
**Uniformly ^15^N-labeled rNTPs and Amidites**		
rNTP (N = A, C, G, or U)	900	CIL
A, or G, or C, or U	1400	Silantes
**Selectively ^15^N-labeled Amidites**		
[1-^15^N]-A	1000	INNotope
[1-^15^N]-G	1100	INNotope
[3-^15^N]-C- or U	1000	Silantes
[1,3-^15^N_2_]-C	1200	Silantes
[1,3,4-^15^N_3_]-C	1000	INNotope
[1,3-^15^N_2_]-U	1000	INNotope
**Uniformly ^2^H/^15^N-labeled rNTPs**		
rNTP (N = A, C, G, or U)	5600	Silantes
**Uniformly ^13^C/^15^N-labeled rNTPs and Amidites**		
rNTP (N = A, C, G, or U)	1100	CIL
N (N = A, C, G, or U Amidites)	5300	Silantes

^a^ Price of rNTPs (CIL, per 100 μmol or Silantes, per 50 mg) and amidites (Silantes or INNotope, per 50 mg) were accessed from their respective websites in July 2021. ^b^ While some of the labeled material is available from multiple suppliers, all prices reflect the cheapest available and are rounded up to the nearest $100.

**Table 2:** Enzymes of the purine de novo biosynthetic pathway.

Enzyme ^a^	Gene	EC Number	PDB ID	Organism ^b^
Hexokinase	hxk1/2	2.7.1.1	1HKG	*Saccharomyces cerevisiae*
Glucokinase	glk	2.7.1.2	1Q18	*Escherichia coli*
Glucose-6-phosphate dehydrogenase	zwf1	1.1.1.49	2BHL	*Homo sapiens*
Phosphogluconate dehydrogenase	gndA	1.1.1.44	2ZYA	*Escherichia coli K-12*
Ribose-5-phosphate isomerase	rpiA	5.3.1.6	1O8B	*Escherichia coli*
Ribose-phosphate diphosphate kinase	prsA	2.7.6.1	3Q89	*Staphylococcus aureus*
Amido phosphoribosyl-transferase	purF	2.4.2.14	IECF	*Escherichia coli*
Phosphoribosylamine-glycine ligase	purD	6.3.4.13	5VEV	*Neisseria gonnorhea*
Phosphoribosylglycinamide formyltransferase	purN	2.1.2.2	3P9X	*Bacillus halodurans*
Phosphoribosylformylglycinamidine synthase	purL	6.3.5.3	1VK3	*Thermotoga maritima*
Phosphoribosylformylglycinamidine cyclo-ligase	purM	6.3.3.1	5VK4	*Neisseria gonorrhoea*
Phosphoribosylamino-imidazole carboxylase (catalytic subunit)	purE	4.1.1.21	4GRD	*Burkholderia cenocepacia*
Phosphoribosylamino-imidazole carboxylase (ATPase subunit)	purK	4.1.1.21	2Z04	*Aquifex aeolicus*
Phosphoribosylamino-imidazole-succinocarboxamide synthase	purC	6.3.2.6	3NUA	*Clostridium perfringens*
Adenylosuccinate lyase	purB	4.3.2.2	3GZH	*Escherichia coli*
Phosphoribosylamino-imidazole-carboxamide formyltransferase	purH	2.1.2.3	1ZCZ	*Thermotoga maritima*
Inosine-monophosphate cyclohydrolase	purH	3.5.4.10	2IU0	*Gallus gallus*
Adenylosuccinate synthase	purA	6.3.4.4	2J91	*Homo sapiens*
Inosine-monophosphate dehydrogenase	guaB	1.1.1.205	1B30	*Homo sapiens*
Guanosine-monophosphate synthase	guaA	6.3.5.2	2YWB	*Thermus thermophilus*
Adenylate kinase	plsA	2.7.4.3	1E4Y	*Escherichia coli*
Creatine phosphokinase	ckmT	2.7.3.2	2CRK	*Oryctolagus cuniculus*
Guanylate kinase	spoR	2.7.4.8	2ANC	*Escherichia coli*
Glycine hydroxymethyltransferase	glyA	2.1.2.1	5VMB	*Acinetobacter baumannii*
Methylene-tetrahydrofolate dehydrogenase	folD	1.5.1.5	1B0A	*Escherichia coli K-12*
Methenyl-tetrahydrofolate cyclohydrolase	folD	3.5.4.9	5TCA	*Homo sapiens*
Aspartate ammonia-lyase	aspA	4.3.1.1	1JSW	*Escherichia coli*
Glutamate dehydrogenase (NAD(P)+)	glud1/ghA	1.4.1.3	4fcc	*Escherichia coli*
Glutamine syntethase	glnA	6.3.1.2	4IS4	*Medicago truncatula*
Inorganic diphosphatase	ppa	3.6.1.1	1IPW	*Escherichia coli*

^a^ All enzymes are commercially available except phosphoribosylformylglycinamidine cyclo-ligase, phosphoribosylamino-imidazole carboxylase, phosphoribosylamino-imidazole-carboxamide formyltransferase, inosine-monophosphate cyclohydrolase, and methenyl-tetrahydrofolate cyclohydrolase. ^b^ Organisms are the sources for the enzymes whose structures are represented by the PDB IDs.

**Table 3:** Enzymes involved in IVT and DNA and RNA ligation.

Enzyme ^a^	Gene	EC Number	PDB ID	Availability	Organism ^a^
T7 RNA polymerase	1	2.7.7.6	1ARO	Thermo Fisher Scientific	*Escherichia coli*
T4 DNA ligase	1.3	6.5.1.1	5WFY	Sigma-Aldrich	*Escherichia coli*
T4 Polynucleotide kinase	pseT	2.7.1.78	1LY1	Thermo Fisher Scientific	*Escherichia coli*
T4 RNA ligase	63	6.5.1.3	5TT6	Thermo Fisher Scientific	*Escherichia coli*
Alkaline phosphatase	ALPL	3.1.3.1	1K7H	New England Biolabs	*Pandalus borealis*

^a^ Organisms are the sources for enzymes whose structures are represented by the PDB IDs.
